# Correction: *Neck circumference and clustered cardiovascular risk factors in children and adolescents: cross-sectional study*

**DOI:** 10.1136/bmjopen-2017-016048corr1

**Published:** 2018-01-09

Castro-Piñero J, Delgado-Alfonso A, Gracia-Marco L The UP&DOWN Study Group, *et al*. Neck circumference and clustered cardiovascular risk factors in children and adolescents: cross-sectional study. *BMJ Open* 2017;7:e016048. doi:10.1136/bmjopen-2017-016048

An error in translating the results from the tables to the text has been included in this study. Statistical analysis was correct as well as the results shown in tables and figure. The correction of this error does not change the results or conclusions of the study, but for clarification, the following corrections are noted:

1. In Results section of the Abstract, ‘NC was negatively associated with maximum oxygen consumption (R2=0.231, P<0.001 for boys; R2=0.018, P<0.001 for girls) and positively associated with SBP, DBP, TC/HDL-c, TG, HOMA, complement factors C-3 and C-4, leptin, adiponectin and clustered CVD risk factor in both sexes (R2 from 0.035 to 0.353, P<0.01 for boys; R2 from 0.024 to 0.215, P<0.001 for girls). Moreover, NC was positively associated with serum C reactive protein, LDL-c and visfatin only in boys (R2 from 0.013 to 0.107, P<0.05).’ should read ‘NC was negatively associated with maximum oxygen consumption (R2=0.231, P<0.001 for boys; R2=0.018, P<0.001 for girls) and adiponectin (R2=0.049, P<0.001 for boys; R2=0.036, P<0.001 for girls); and positively associated with SBP, DBP, TC/HDL-c, TG, HOMA, complement factors C-3 and C-4, leptin and clustered CVD risk factor in both sexes (R2 from 0.035 to 0.353, P<0.01 for boys; R2 from 0.024 to 0.215, P<0.001 for girls). Moreover, NC was positively associated with serum C reactive protein and LDL-c only in boys (R2 from 0.013 to 0.055, P<0.05)’.
2. In Clustered CVD risk factors measurement section of the Method, ‘The VO2max z-score was inverted, because higher cardiorespiratory fitness is associated with lower fatness’ should read ‘The VO2max and adiponectin z-scores were inverted, because higher values of both are associated with lower fatness’.
3. Related to Table 3 of the Results, ‘More specifically, NC was negatively correlated with VO2max (r=−0.481, P<0.001 for boys; r=−0.672, P<0.001 for girls), positively correlated with SBP, DBP, TG, HOMA, C-3, C-4, leptin and adiponectin in both sexes (r from 0.167 to 0.419; all, P<0.01), and with TC/HDL-c, LDL-c, CRP and visfatin only in boys (r from 0.243 to 0.388; all, P<0.001).’ should read ‘More specifically, NC was negatively correlated with VO2max (r=−0.481, P<0.001 for boys; r=−0.672, P<0.001 for girls), adiponectin (r=−0.228, P<0.001 for boys; r=−0.198, P<0.001 for girls) and visfatin only in boys (r=−0.338, P<0.001), and positively correlated with SBP, DBP, TG, HOMA, C-3, C-4 and leptin in both sexes (r from 0.167 to 0.419; all, P<0.01), and with TC/HDL-c, LDL-c and CRP only in boys (r from 0.243 to 0.388; all, P<0.001)’.
4. Related to Table 4 of the Results, ‘NC was negatively associated with VO2max (R2=0.231, P<0.001 for boys; R2=0.018, P<0.001 for girls), and positively associated with SBP, DBP, TC/HDL-c, TG, HOMA, C-3, C-4, leptin, adiponectin and clustered CVD risk factor in both sexes (R2 from 0.035 to 0.353, P<0.01 for boys; R2 from 0.024 to 0.215, P<0.001 for girls). Moreover, NC was positively associated with CRP, LDL-c and visfatin only in boys (R2 from 0.013 to 0.107, P<0.05).’ should read ‘NC was negatively associated with maximum oxygen consumption (R2=0.231, P<0.001 for boys; R2=0.018, P<0.001 for girls), adiponectin (R2=0.049, P<0.001 for boys; R2=0.036, P<0.001 for girls) and visfatin only in boys (R2=0.107, P<0.001); and positively associated with SBP, DBP, TC/HDL-c, TG, HOMA, complement factors C-3 and C-4, leptin and clustered CVD risk factor in both sexes (R2 from 0.035 to 0.353, P<0.01 for boys; R2 from 0.024 to 0.215, P<0.001 for girls). Moreover, NC was positively associated with CRP and LDL-c only in boys (R2 from 0.013 to 0.055, P<0.05)’
5. In NC and single CVD risk factors section of Discussion ‘In the present study, IL-6 and visfatin were associated with WHtR and FMI in both sexes and only with NC in boys’ should read ‘In the present study, IL-6 was associated with WHtR and FMI in boys only, whilst visfatin was associated with NC in boys only’

